# Establishing a tree shrew model of systemic lupus erythematosus and cell transplantation treatment

**DOI:** 10.1186/s13287-016-0385-1

**Published:** 2016-08-24

**Authors:** Guang-Ping Ruan, Xiang Yao, Ju-Fen Liu, Jie He, Zi-An Li, Jian-Yong Yang, Rong-Qing Pang, Xing-Hua Pan

**Affiliations:** 1 The Cell Biological Therapy Center, Kunming General Hospital of Chengdu Military Command, Kunming, 650032 People’s Republic of China; 2 Stem Cells and Immune Cells Biomedical Techniques Integrated Engineering Laboratory of State and Regions (Yunnan Province), Kunming, 650032 People’s Republic of China; 3 Cell Therapy Technology Transfer Medical Key Laboratory of Yunnan Province, Kunming, 650032 People’s Republic of China

**Keywords:** Systemic lupus erythematosus, Tree shrews, Animal models, Umbilical cord mesenchymal stem cells, Transplantation

## Abstract

**Background:**

The establishment of a tree shrew model for systemic lupus erythematosus (SLE) provides a new method to evaluate the pathogenesis of autoimmune diseases.

**Methods:**

Eighty tree shrews were randomly divided into four groups receiving either an intraperitoneal injection of pristane, lipopolysaccharide (LPS), or pristane and LPS, or no injection. Three weeks after injection, the SLE model tree shrews were divided into the model group and the treatment group. Tree shrews in the treatment group and the normal control group were infused with umbilical cord mesenchymal stem cells (UC-MSCs). The cells were labeled with DiR. Two weeks after transplantation, three groups of tree shrews were analyzed for urine protein, serum antinuclear antibodies and antiphospholipid, and inflammatory cytokine antibody microarray detection. The heart, liver, spleen, lung, and kidney were collected from the three groups and subjected to hematoxylin and eosin (HE) staining and detection of renal immune complex deposition.

**Results:**

HE staining indicated pathology in the model group. Red fluorescence revealed immune complex deposition in the kidneys from the model group.

**Conclusions:**

The combined intraperitoneal injection of pristane and LPS is the best way to induce SLE pathological changes. The pathological changes improved after UC-MSC treatment.

**Electronic supplementary material:**

The online version of this article (doi:10.1186/s13287-016-0385-1) contains supplementary material, which is available to authorized users.

## Background

Systemic lupus erythematosus (SLE) is an autoimmune disease of unknown etiology. A variety of autoantibodies and immune complexes appear in the serum of SLE patients, along with severe glomerulonephritis [1]. Effective treatments for SLE are presently lacking. The establishment of an appropriate animal model is an essential research tool to investigate drug treatment efficacy during the pathogenesis of the disease. Tree shrews are climbing animals between insectivores and primates. Evolutionarily closer to primates than rodents, tree shrews are being considered as an alternative to primates for study. Because tree shrews are small in size and cheap in price compared with primates, we chose this model over primates. Tree shrews were found to have genetic characteristics close to those of primates, which laid the foundation for research into clinical disease mechanisms and the development of new drugs.

The kidney is the most affected organ in mouse and human SLE. Lupus nephritis results in immune complex deposition in different parts of the glomeruli [2]. When lupus nephritis cannot be well controlled, glomerulonephritis will eventually develop and advance to renal failure [3]. Generally, SLE therapy – particularly cyclophosphamide therapy – can control most lupus nephritis well, but not all cases can be controlled. Better understanding of the pathogenesis of the disease is thus very important to improve the therapeutic effect of SLE. SLE has a complex etiology, and the exact mechanism of this autoimmune disease has not been elucidated. However, SLE is generally agreed to be due to a genetic predisposition and appears in response to environmental factors and infections. Specifically, a susceptibility gene regulates the pathogenesis of SLE, and lymphocytes and cytokines participate in abnormal immune responses, which significantly increase autoantibodies and damage to the body.

Mesenchymal stem cells (MSCs) are a class of cells found in the bone marrow, fat, muscle, and other tissues. These cells are multipotent and have the immunomodulatory effects of nonhematopoietic stem cells [4]. Adult bone marrow mesenchymal stem cells (BM-MSCs) mainly form the hematopoietic microenvironment and regulate the growth and differentiation of hematopoietic stem cells. In addition, MSCs can differentiate into bone, cartilage, fat, cardiac tissue, neuronal tissue, and other mesodermal cells. MSCs have low or no immunogenicity [5], which decreases the probability of allogeneic transplant rejection and thus constitutes safer treatment. Umbilical cord mesenchymal stem cells (UC-MSCs) have the advantage of easier access than the BM-MSCs used in the past, which has made them a clinical research focus [6].

SLE occurs mainly in young women and is an autoimmune disease that involves multiple organs and is characterized by T-cell and B-cell activation. The exact mechanism of the pathogenesis of SLE remains unclear, but studies have confirmed that abnormal T-cell immunity plays an important role. This abnormal immunity strongly relates to the development and prognosis of the disease. T-helper 17 (Th17) cells are novel CD4^+^ effector T cells identified in recent years that may secrete interleukin (IL)-17, which is involved in the innate immune and adaptive immune responses and plays an important role in the pathogenesis of SLE [7].

Furthermore, IL-17 is involved in the innate immune response and induces expression of inflammatory cytokines IL-6 and IL-16, among others; amplifies tumor necrosis factor alpha (TNFα) during inflammation; and is involved in adaptive immune responses. Studies have shown that Th17 cells are involved in the pathogenesis of SLE [8], rheumatoid arthritis (RA), autoimmune encephalomyelitis (EAE), autoimmune enteropathy, psoriasis, and other diseases. The transplantation of UC-MSCs reduces the levels of Th17 cells in SLE patients [9], and a decrease in Th17 lymphocytes is likely one of the effective mechanisms of UC-MSC transplantation in the treatment of SLE [10].

In recent years, the incidence of autoimmune disease has increased and SLE remains a problem. When studying this topic, an animal model of SLE is one of the best research tools, especially for the study of the genetic aspects of susceptibility and pathogenesis. The use of the tree shrew model can control the impact of environmental factors on experimental animals, help to clarify the pathogenesis of the disease, and help to identify the pathogenic gene locus. The tree shrew model developed in this study aims to elucidate the exact mechanism of lupus nephritis and the mechanisms and efficacy of UC-MSCs in the treatment of SLE.

## Methods

### Tree shrew UC-MSCs isolated, cultured, and identified

Full-term pregnant tree shrews were anesthetized using intraperitoneal pentobarbital sodium (50 mg/kg body weight) before caesarean section. The uterus was first surgically opened to fully expose the placenta and the fetal tree shrews, and approximately 5 cm of umbilical cord was isolated from the fetus and placenta via sterile laparotomy; approximately three or four umbilical cords were isolated based on the number of fetuses. The umbilical cord was cleaned on a sterile bench and soaked with double antibiotics. The umbilical cord was then cut and placed in adherent culture flasks, in which adherent cells were visible after 2–3 days. The medium was 20 % fetal bovine serum in DMEM-F12 (Hyclone). The cells were subcultured when confluent. The third passages of tree shrew UC-MSCs were labeled with antibodies for flow cytometry. The antibodies included CD29-FITC, CD44-FITC, CD34-FITC, and FITC isotype controls. CD antibodies were purchased from BD Company. Experimental protocols were approved by the Experimental Animal Ethics Committee of Kunming General Hospital of Chengdu Military Command.

### Tree shrew grouping and injection

Eighty *Tupaia belangeri* Chinese tree shrews that had been domesticated by the Institute of Medical Biology, Chinese Academy of Medical Sciences at the Tree Shrew Germplasm Resource Center were randomly divided into four groups of 20. The groups received one of the following treatments: intraperitoneal injection of 1 ml pristane, intraperitoneal injection of 1 ml lipopolysaccharide (LPS), intraperitoneal injection with pristane and LPS, and no injection (normal controls). Pristane and LPS were purchased from Sigma Chemical Co.; LPS was dissolved to 0.5 mg/ml, and the injection volume was 1 ml per tree shrew. LPS and pristane were injected once every week for 3 weeks. After injection for 1, 2, or 3 weeks, the serum was collected and packaged in an ELISA plate. HRP-labeled rabbit anti-monkey IgG antibody was used to observe serum IgG changes. Each tree shrew serum sample was then sent to a clinical laboratory to detect complement C3 levels.

### Quantitative PCR

Blood (0.5 ml) was collected from all tree shrews in each group. RNA was extracted using a blood RNA extraction kit from Baitaike according to the manufacturer’s instructions. Reverse transcription was carried out using the reverse transcription kit from Thermo according to the manufacturer’s instructions. Quantitative PCR was carried out using Thermo quantitative PCR reagents to detect the relative expression of IL-17 and Foxp3. The primer sequences and product lengths are presented in Table 1. The relative expression of IL-17 and Foxp3 was normalized by comparison with *GAPDH*.

**Table 1:** Primer sequences and product lengths

Gene	Primer sequence	Product length (base pairs)	Annealing temperature (°C)
*IL-17*	TATGAGGAGCAAATGGGTCA	117	60
	CAGCAAAGTAACATCCAGCCTA		
*Foxp3*	CACTCAAGGAGGCGTTGTC	175	60
	GGTGGCATAGGTGAAAGGAG		
*GAPDH*	GTTTGTGATGGGCGTGAAC	171	60
	GTCTTCTGGGTGGCAGTGA		

### ELISA detection of tree shrew serum IgG

Because monkey secondary antibody reagents cross-react with tree shrew IgG, we purchased an HRP-labeled rabbit anti-monkey IgG antibody (Sigma) to detect tree shrew serum IgG. The serum was packaged in an ELISA plate at 4 °C overnight and then incubated with HRP-labeled rabbit anti-monkey IgG antibody at 37 °C for 1 hour and washed three times. The chromogenic agent TMB was used to obtain a colorimetric optical density (OD) value at 450 nm.

### Model tree shrew grouping

According to the results of quantitative PCR and serum IgG ELISA, 10 modeled tree shrews were selected from the pristane and LPS group. The ELISA OD of standard model tree shrews was >2.8, more than twice the mean OD value (1.4) of the normal control group. Quantitative PCR showed that the relative expression of the *IL-17* gene was more than twice that of the normal control group, while the relative expression of the *Foxp3* gene was less than 0.5 that of the normal control group.

### Labeling and transplantation of tree shrew UC-MSCs

Ten model tree shrews were divided into the model control group and the treatment group with five animals per group, and five normal tree shrews were then randomly selected as the normal control group. The UC-MSCs of tree shrews were digested with 0.25 % trypsin, and the digestion was terminated with complete medium containing 20 % FBS. The cells were uniformly pipetted, aspirated into a 15 ml centrifuge tube, and counted. The cells were labeled at a concentration of 1 × 10^6^ cells/ml, and 1 ml of this cell suspension was added to 5 μl of a 3 mM stock solution of DiR. The resulting mixture was incubated at 37 °C for 10 minutes and then washed three times with prewarmed serum-free medium (centrifugal rotation: 2000 rev/min, centrifugation time: 5 minutes). The labeled cells (1 × 10^6^ cells) were injected into the tail veins of treatment group and normal control group animals.

### ELISA detection of serum antiphospholipid and antinuclear antibodies

Two weeks after cell transplantation, venous blood was collected from three groups of tree shrews. The serum was separated to detect antiphospholipid and antinuclear antibody changes. The antiphospholipid ELISA kit was purchased from Abcam Company and the antinuclear antibody ELISA kit was purchased from ALPHA DIAGNOSTIC Company. The operating steps were followed strictly according to kit instructions.

### Three groups of tree shrews: urinary protein quantitation

Two weeks after cell transplantation, tree shrew morning urine was collected from three groups. The urinary protein concentration was detected by the Bradford method. The protein assay kit was purchased from Biyuntian Company. The steps were followed in strict accordance with the kit instructions.

### Three groups of tree shrews: serum inflammatory cytokine antibody microarray analysis

Two weeks after cell transplantation, venous blood was collected from three groups of tree shrews. Serum was separated to detect inflammatory cytokine antibodies by microarray. The chips were purchased from Raybiotech Company. The detection steps were followed strictly according to the instructions.

### HE staining and kidney Masson and PAS staining

Two weeks after cell transplantation, the heart, liver, spleen, lung, and kidney of the normal control group, the model control group, and the treated group were soaked in 4 % paraformaldehyde and sent to Wuhan Google Biotechnology Co., Ltd for hematoxylin and eosin (HE) staining and kidney Masson and periodic acid–Schiff (PAS) staining. The stained sections were photographed and analyzed. From each section within each group, three images were randomly selected and photographed at 400× magnification for analysis. The Image-Pro Plus6.0 software tool was used to circle each glomerular picture. The analysis of each glomerulus from each photograph shows the red sugar area ratio of the total area of the glomerulus, which is the percentage of carbohydrate area.

### Detection of renal immune complex deposition

The sections were deparaffinized in xylene solution and subjected to antigen retrieval. The sections were first incubated with rabbit anti-monkey IgG antibody at 4 °C overnight, followed by a solution of secondary antibody (CY3; goat anti-rabbit 1:300), which covered the tissue at room temperature in the dark for 60 minutes. DAPI-stained nuclei slices were mounted and photographed under a microscope.

### Imaging experiments

Two weeks after cell transplantation, two animals from the treatment group, the normal control group, and the model group were euthanized, and the heart, liver, spleen, lung, and kidney were subjected to imaging. Each organ was placed in a dish for in-vivo imaging with a small animal instrument to observe the distribution of DiR-labeled cells.

### Statistical analysis

The data are shown as the mean ± SD. The groups were compared using one-way ANOVA with SPSS 17.0 statistical software. *P* < 0.05 was considered statistically significant.

## Results

### Characterization of isolated UC-MSCs

**Fig. 1:**
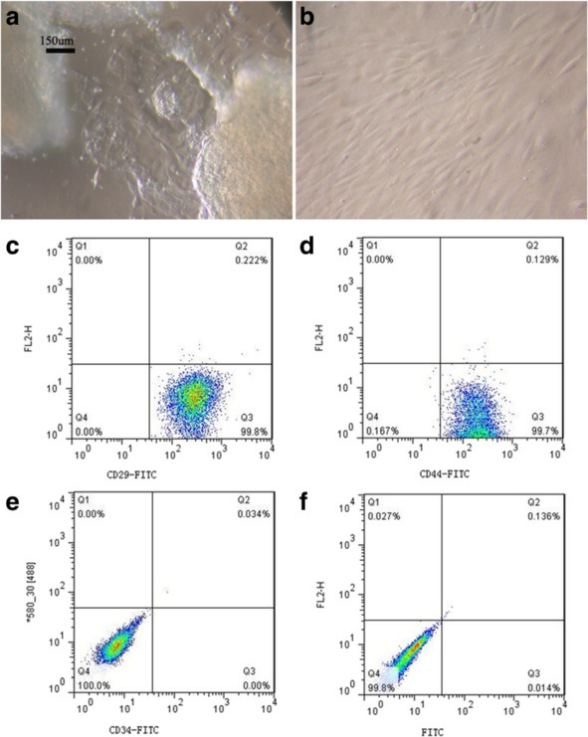
Characterization of isolated tree shrew UC-MSCs. **a** Tree shrew UC-MSCs were just adherent. **b** Tree shrew UC-MSCs covered to be passaged. **c** CD29-FITC-positive rate of 99.8 % because UC-MSCs were positive for expression with CD29. **d** CD44-FITC-positive rate of 99.7 % because UC-MSCs were positive for expression with CD44. **e** CD34-FITC is negative because UC-MSCs were negative for expression with CD34. **f** Isotype control is negative. The results showed that the UC-MSCs of the tree shrews separated and cultured had UC-MSC surface markers

After 3 days in adherent culture, many UC-MSCs survived. After the medium was changed three times, the cells were evenly distributed and fusiform, and could be passaged after confluence. Flow cytometry results showed a CD29-FITC-positive rate of 99.8 %, a CD44-FITC-positive rate of 99.7 %, a CD34-FITC-positive rate of 0 %, and a FITC isotype control rate of 0.014 %. These results showed that the UC-MSCs of the tree shrews separated and cultured had MSC surface markers (Fig. 1).

### Quantitative PCR results

**Fig. 2:**
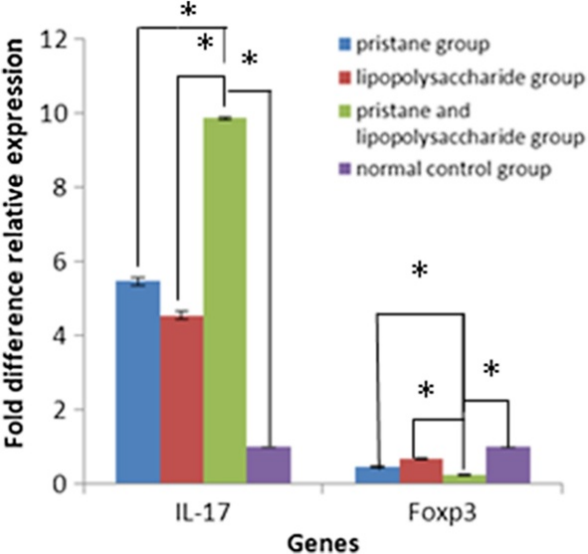
Gene expression levels in peripheral blood based on quantitative PCR (*n* = 20). Relative expression levels of the control group were set to 1, and *IL-17* expression levels in the LPS and pristane, the pristane only, and the LPS only groups are higher than in the control group (*P < 0.01), and *IL-17* expression levels in the LPS and pristane group are significantly higher than in the pristane only and LPS only groups (*P < 0.01 compared with the other three groups, *n* = 20). On the contrary, *Foxp3* expression levels in the LPS and pristane, the pristane only, and the LPS only groups are lower than in the control group (*P < 0.01), and *Foxp3* expression levels in the LPS and pristane group are significantly lower than in the pristane only and LPS only groups (*P < 0.01 compared with the other three groups, *n* = 20)

Figure 2 shows that, 2 weeks after the injection of pristane and LPS, the *IL-17* expression levels in the LPS and pristane, the pristane only, and the LPS only groups are higher than in the control group (*P* < 0.01), and that *IL-17* expression levels in the LPS and pristane group are significantly higher than in the pristane only and LPS only groups (*P* < 0.01 compared with the other three groups, *n* = 20). On the contrary, *Foxp3* expression levels in the LPS and pristane, the pristane only, and the LPS only groups are lower than in the control group (*P* < 0.01), and the *Foxp3* expression levels in the LPS and pristane group are significantly lower than in the pristane only and LPS only groups (*P* < 0.01 compared with the other three groups, *n* = 20).

### ELISA to detect changes in serum IgG and complement C3 level in tree shrews

**Fig. 3:**
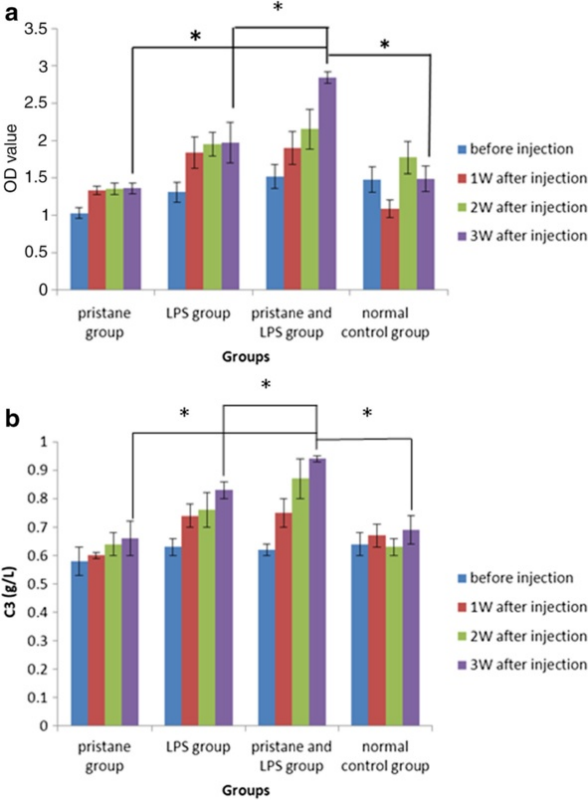
ELISA was used to detect changes in the tree shrew serum IgG and complement C3 levels at 1, 2, and 3 weeks after injection (*n* = 20) (*P < 0.01 compared with the other groups 3 weeks after injection). **a** Serum IgG levels of tree shrews injected with pristane and LPS increased most significantly 3 weeks after injection (*P < 0.01 compared with the other three groups, *n* = 20). **b** Complement C3 levels increased most significantly in the pristane and LPS group 3 weeks after injection (*P < 0.01 compared with the other three groups, *n* = 20). *LPS* lipopolysaccharide, *OD* optical density, *W* week

The ELISA test results showed that serum IgG levels of tree shrews injected with pristane and LPS increased most significantly 3 weeks after injection (*P* < 0.01 compared with the other three groups, *n* = 20) (Fig. 3a). The complement C3 levels increased most significantly in the pristane and LPS group 3 weeks after injection (*P* < 0.01 compared with the other three groups, *n* = 20) (Fig. 3b). The ELISA OD of standard model tree shrews was >2.8, more than twice the mean OD value (1.4) of the normal control group. Quantitative PCR showed that the relative expression of the *IL-17* gene was more than twice that of the normal control group, while the relative expression of the *Foxp3* gene was less than 0.5 that of the normal control group. Ten tree shrews were selected from the pristane and LPS group, divided into a model control group and a treatment group (each with five animals). Five normal tree shrews were then randomly selected as the normal control group. In the treatment and normal control groups, 1 × 10^6^ UC-MSCs from tree shrews labeled with DiR were injected into the tail vein. Two weeks after transplantation, the organs of each group were subjected to imaging and HE staining.

### Results of antiphospholipid and antinuclear antibody, and urine protein

**Fig. 4:**
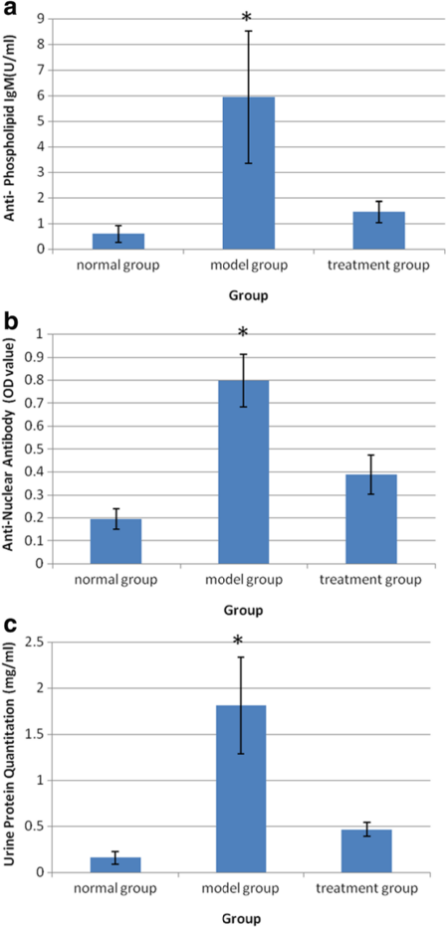
Antiphospholipid and antinuclear antibody and urine protein. **a** ELISA results of antiphospholipid showed that levels of serum antiphospholipid IgM (U/ml) were significantly higher in the model group (*P < 0.01 compared with the other two groups, *n* = 5). **b** ELISA results of antinuclear antibody showed that levels of serum antinuclear antibodies in the model group were significantly higher (*P < 0.01 in comparison with the other two groups, *n* = 5). **c** Urine protein quantitation results in the model group were significantly increased (*P < 0.01 in comparison with the other two groups) and the urinary protein was significantly decreased in the treatment group (*n* = 5). *OD* optical density

Antiphospholipid test results showed that levels of serum antiphospholipid IgM (U/ml) were significantly higher in the model group (*P* < 0.01 compared with the other two groups, *n* = 5). In the normal group, serum anti-phospholipid IgM was low and antiphospholipid IgM in the treatment group was significantly decreased (Fig. 4a). Antinuclear antibody test results showed that levels of serum antinuclear antibodies in the model group were significantly higher (*P* <0.01 in comparison with the other two groups, *n* = 5), the normal group serum anti-nuclear antibody OD value was lower, and the antinuclear antibody level of the treatment group was significantly lower (Fig. 4b). Urinary protein results showed that the mean urinary protein concentration was 1.812 mg/ml in the model group (*n* = 5), the mean urinary protein concentration of the normal group was 0.1554 mg/ml, and the mean urinary protein concentration of the treatment group was 0.462 mg/ml. Urinary protein concentrations in the model group were significantly increased (*P* < 0.01 in comparison with the other two groups, *n* = 5) and the urinary protein was significantly decreased in the treatment group (Fig. 4c).

### Inflammatory cytokine antibody microarray results

**Fig. 5:**
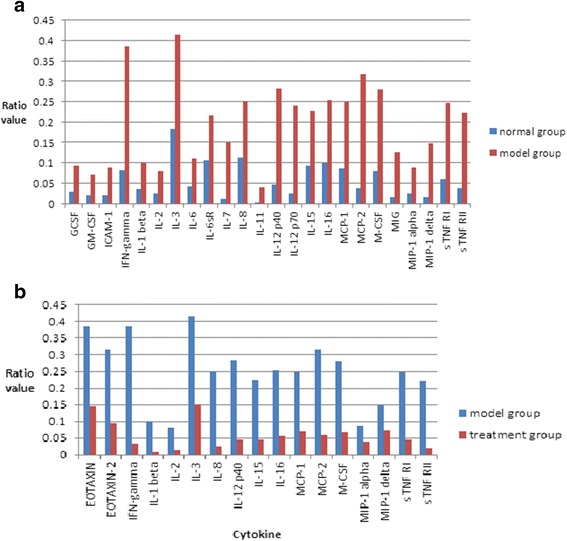
Inflammatory cytokine antibody microarray results. **a** Model group vs normal group; 24 inflammatory cytokines increased significantly. The upregulated rate is more than 2.0. **b** Treatment group vs model group; 17 inflammatory cytokines decreased significantly. The downregulated rate is less than 0.5

Microarray results showed that 24 inflammatory cytokines increased significantly in the model group vs the normal group. The upregulated rate is more than 2.0 (Fig. 5a). In the treatment group vs the model group, 17 inflammatory cytokines decreased significantly. The downregulated rate is less than 0.5 (Fig. 5b). In the model group, many inflammatory cytokines increased significantly compared with the normal group; while in the treatment group, many inflammatory cytokines decreased significantly compared with the model group.

### HE staining of organs and Masson and PAS staining of kidney

**Fig. 6:**
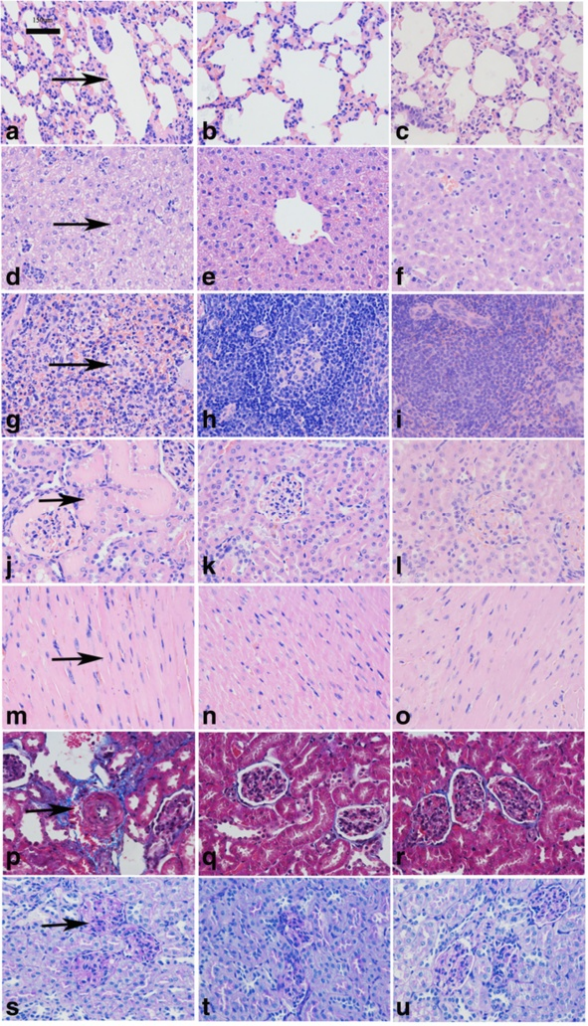
HE staining and renal Masson and PAS staining for three groups of tree shrew organs (400× magnification). **a**–**o** HE staining: **a** model control group showing slightly thickened alveolar septa and some alveolar atrophy, alveolar compensatory expansion, and bulla formation (*arrow*); **b** normal control group with normal lung; **c** treatment group without bulla formation. **d** model control group with diffuse hepatic cell necrosis formation (*arrow*) and eosinophilic bodies, granulocyte infiltration, and a large amount of liver cell degeneration; **e** normal control group with normal liver; **f** treatment group without granulocyte infiltration; **g** model control group showing a large amount of granulocyte infiltration in spleen red pulp (*arrow*); **h** normal control group with normal spleen; **i** treatment group without granulocyte infiltration; **j** model control group with part of the renal cyst and renal tubular cavity showing a large lightly stained mucus-like substance (*arrow*); **k** normal control group with normal kidney; **l** treatment group without mucus-like substance; **m** model control group with minor bleeding and a small amount of cardiac hypertrophy (*arrow*); **n** normal control group with normal heart; and **o** treatment group returned to normal without cardiac hypertrophy. **p**–**r** Kidney Masson staining: **p** model control group with renal interstitial fibrosis (*arrow*; fibrosis in *blue*); **q** normal control group with normal kidney; and **r** treatment group with less renal interstitial fibrosis. **s**–**u** Kidney PAS staining: **s** model control group with glycogen deposition in glomeruli (*arrow*; glycogen dyed *red–purple*); **t** normal control group with less glycogen deposition; and **u** treatment group with less glycogen deposition (Color figure online)

Two weeks after transplantation with tree shrew UC-MSCs, the HE staining results showed that the lung (Fig. 6a), liver (Fig. 6d), spleen (Fig. 6g), kidney (Fig. 6j), and heart (Fig. 6m) of the model control group experienced varying degrees of disease, while those of the normal control group (Fig. 6b, e, h, k, n) were normal. Compared with the model control group, the lesions of the treatment group significantly improved (Fig. 6c, f, i, l, o). The kidney Masson staining of the model control group showed renal interstitial fibrosis (Fig. 6p), while fibrosis was absent in the normal control group (Fig. 6q) and the treatment group returned to normal (Fig. 6r). Fibrosis is shown in blue. The kidney PAS staining of the model control group showed red carbohydrate deposition in the glomeruli (Fig. 6s), but glomerular red carbohydrate deposition was reduced in the normal control group (Fig. 6t) and the treatment group (Fig. 6u). Glycogen dyed red–purple. The percentage of carbohydrate area for the three groups was analyzed. The results are summarized in Table 2 (*P* < 0.05 compared with the other two groups, *n* = 5). Statistical analysis showed that the percentage of sugar area in the model group was significantly higher than in the normal group and the treatment group. There was no significant difference between the normal group and the treatment group, indicating that the occurrence of renal lesions improved after treatment, returning to near normal. Ten sections of each organ in each group and 10 visual fields from each section were analyzed. In total, 100 visual fields from each group were analyzed and the results are summarized from more than 60 visual fields with similar results. The similar results are shown in one picture in Fig. 6.

**Table 2:** Comparison of sugar area percentage revealed by renal PAS staining in three groups

	Normal control group	Treatment group	Model control group*
Sugar area percentage	2.45 ± 1.08	4.48 ± 0.74	18.55 ± 3.48

*P < 0.05 compared with the other two groups (*n* = 5)

*PAS* periodic acid–Schiff

### Detection of renal immune complex deposition

**Fig. 7:**
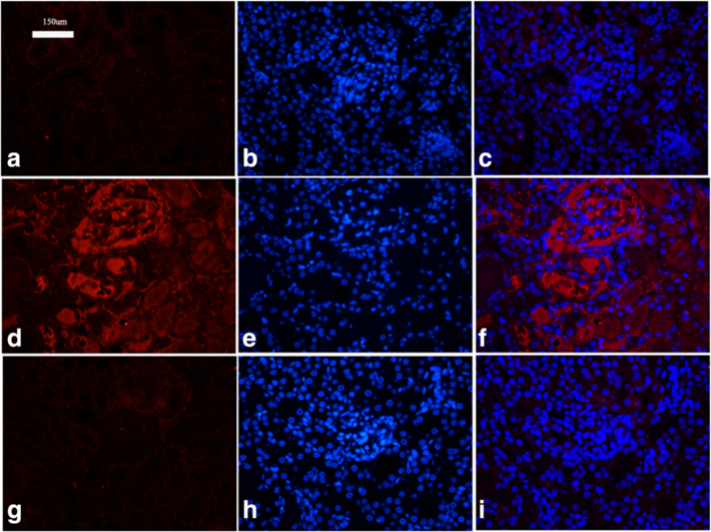
Detection of renal immune complex deposition. **a**–**c** Normal control group: the kidney has no immune complex deposition (*no red fluorescence*). **d**–**f** Model control group: the kidney has plenty of immune complex deposition (*red fluorescence* shows immune complex deposition). **g**–**i** Treatment group: the kidney has less immune complex deposition (*less red fluorescence*). **a**, **d**, **g** Red fluorescence channel. **b**, **e**, **h** Blue fluorescence channel, DAPI staining. **c**, **f**, **i** Merged red and blue fluorescence channels (Color figure online)

The kidney sections were incubated with rabbit anti-monkey IgG. The results show a large number of immune complexes deposited in the kidneys of the model group (Fig. 7d, f); fluorescence substances had been deposited, but not in the normal control group (Fig. 7a, c). The treatment group had fewer renal immune complex depositions (Fig. 7h, j). These results indicate that renal immune complex depositions reduced after treatment.

### Imaging results

**Fig. 8:**
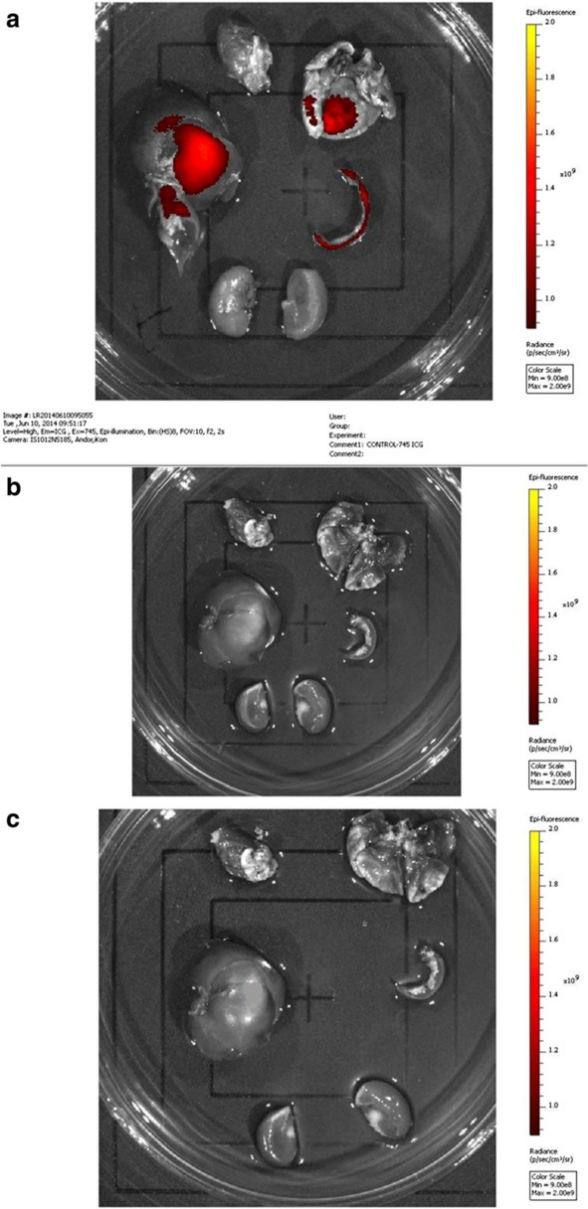
Imaging of tree shrew organs. **a** Transplanted labeled cells in the treatment group: labeled cells distributed to the lung, liver, and spleen. **b** Transplanted labeled cells in the normal control group: labeled cells did not distribute. **c** Untreated model control group: labeled cells were not detected. The fluorescence intensity values of the treatment group were significantly higher than those of the normal control group and the model control group

Two weeks after cell transplantation, blood and urine were collected from the tree shrews in the treatment, normal control, and model control groups. The animals were euthanized and the heart, liver, spleen, lung, and kidney were subjected to imaging. The organs were placed in a Petri dish, and in-vivo small animal imaging devices were used to observe the distribution of DiR-labeled cells (Fig. 8). The labeled cells distributed to the lung, liver, and spleen in the treatment group (Fig. 8a). The labeled cells did not distribute in the normal control (Fig. 8b) and model control (Fig. 8c) groups.

The fluorescence intensity values of various organs are presented in Table 3.

**Table 3:** Fluorescence intensity values of various organs

Organ	Normal control group	Treatment group*	Model control group
Heart	9.324 × 10^8^	2.551 × 10^9^	9.124 × 10^8^
Liver	8.158 × 10^9^	7.101 × 10^10^	8.039 × 10^9^
Kidney	4.719 × 10^9^	9.041 × 10^10^	4.523 × 10^9^
Spleen	6.088 × 10^8^	9.901 × 10^9^	6.901 × 10^8^
Lung	1.941 × 10^9^	3.475 × 10^10^	1.894 × 10^9^

*P < 0.05 compared with the other two groups

The fluorescence intensity values of the treatment group were significantly higher than those of the normal control group and the model control group, and the difference was statistically significant (*P* < 0.05 compared with the other two groups).

## Discussion

SLE incidence is believed to be related to genetic causes, hormonal factors, certain medications, infections, and other factors. Similar to other autoimmune diseases, the body continues to produce IgG class autoantibodies to self-antigens, forming a large number of circulating immune complexes deposited in the glomeruli capillaries and other tissues to cause glomerulonephritis and whole body multiple systemic disease. The mechanism is similar to type III hypersensitivity. Currently, nonspecific immunosuppression is used in clinical treatment, and hematopoietic stem cell transplantation is used to treat a small number of refractory cases. However, SLE prognosis remains to be improved to prevent infection, recurrence, kidney failure, and heart and brain damage, and for other reasons. We hope that the establishment of the tree shrew SLE model and infusion of UC-MSCs in this new therapeutic animal study will help to improve treatment efficacy and explore a therapeutic mechanism.

Under certain conditions, MSCs can differentiate into a variety of tissue cells, such as osteoblasts, chondrocytes, tendon cells, and adipocytes. However, they can also differentiate across the mesoderm to the endoderm and neural ectoderm (such as the liver, bile duct epithelium, lung, intestine, skin epithelium, and neuronal and glial cells) [11]. Pan et al. [12] have reported using BM-MSC transplantation to treat diabetic nephropathy in tree shrews. However, UC-MSCs are readily available and not rejected after replantation; these cells show a strong ability to self-replicate, are easily separated and amplified in vitro, and should be widely used in tissue engineering, cell transplantation, and gene therapy. UC-MSCs are therefore an ideal seed cell. UC-MSCs have immunosuppressive effects. Application of UC-MSC treatment for SLE involves allogeneic cell transplantation. By restoring the balance of autoreactive T cells in SLE patients, UC-MSCs exert immunosuppressive effects by reaching immune homeostasis again and achieve a therapeutic effect.

The role of CD4^+^CD25^+^ T cells is not fully understood [13]. The possible mechanisms are secretion of TGF-β and/or IL-10 or induction of other cells to secrete other inhibitory cytokines. With high cell surface expression of TGF-β, CD4^+^CD25^+^ T cells bind the TGF-β receptor and inhibit the autoreactive T cells and B cells. CD4^+^CD25^+^ T cells in the body account for 10 % of total CD4^+^ T cells. The number and activity of CD4^+^CD25^+^ T cells meet the need to inhibit autoreactive T-lymphocyte and B-lymphocyte activation, as well as the proliferation and maintenance of autoimmune balance. In individuals susceptible to SLE, environmental factors, genetic abnormalities, and other factors affect the production of CD4^+^CD25^+^ T cells in the thymus, which can lead to SLE [14]. Previous studies showed that the level of CD4^+^CD25^+^ T cells was significantly lower in SLE patients than in healthy controls, irrespective of the status (active or inactive) [15]. Because the numbers of CD4^+^CD25^+^ T cells were reduced, which results in immune suppression, the activation of T-helper cells was enhanced, the expression of B-cell activating factor increased, and B-lymphocyte hyperthyroidism was induced to produce a variety of autoantibodies, which led to a series of immune system interaction disorders that resulted in multiple organ damage. We used quantitative PCR to detect the *Foxp3* gene because its change indirectly represents the change in CD4^+^CD25^+^ T cells in peripheral blood. Our results showed in that in the model tree shrew, the expression of the *Foxp3* gene decreased. The relative expression of the *Foxp3* gene was less than 0.5 of that in the normal control group (*P* < 0.01). The decrease in *Foxp3* gene expression is enough to have biological impact on CD4^+^CD25^+^ T cells. This result indirectly shows that in the SLE model CD4^+^CD25^+^ T cells are significantly lower than in healthy controls. According to the literature, CD4^+^CD25^+^ T cells are a subset of regulatory T cells that mainly originate from the thymus. Their main function is related to the inhibition of the immune response of autoreactive T cells, the inhibition of conventional T-cell activation, and the promotion of the secretion of some suppression cytokines. The cells play an important role in the maintenance of a stable internal environment and tumor immune surveillance, and induce transplantation tolerance and autoimmune diseases. SLE patients with increased Th17 cells in the peripheral blood show a decreased population of CD4^+^CD25^+^ T cells, which increases peripheral *IL-17* gene expression and decreases *Foxp3* gene expression.

The literature indicates the use of LPS [16–18] and pristane [19–21] by intraperitoneal injection to induce SLE in mouse studies, but the tree shrew SLE model has not been studied. We therefore used 80 tree shrews, divided into four groups of 20. Studies have shown that pristane and LPS injection in tree shrews induces SLE-related changes, including an increase in the peripheral *IL-17* gene [22], reduction in the *Foxp3* gene, elevated serum IgG and C3, multiorgan lesions, and immune complex deposition in kidney sections. We divided 10 model tree shrews into five model control groups and five treatment groups, and the model success criterion was defined as an ELISA OD value of standard model tree shrews >2.8, more than twice the mean OD value (1.4) of the normal control group. Quantitative PCR showed that the relative expression of the *IL-17* gene was more than twice that of the normal control group, while that of the *Foxp3* gene was less than 0.5 that of the normal control group. DiR-labeled cells (1 × 10^6^ cells) were transplanted into each tree shrew in the treatment and the normal control groups; 2 weeks later, three groups of tree shrews were used to detect serum antiphospholipid and antinuclear antibodies simultaneously with an inflammatory cytokine antibody microarray. From three groups of tree shrews, morning urine was analyzed for urine protein concentration, and the heart, liver, spleen, lungs, and kidneys were imaged. The imaging results showed that labeled cells were distributed in the lung, liver, and spleen of tree shrews in the treatment group, and fluorescence intensity values of the normal and model control groups of all organs were lower than in the treatment group. Labeled UC-MSCs from tree shrews mainly were located in the damaged organs. Because organ damage was absent in the normal control group, the detected fluorescence intensity values were lower than those in the treatment group, and most values were lower by an order of magnitude. Labeled cells did not transplant in the model control group; thus, the detected fluorescence intensity values were lower than in the treatment group, and most values were lower by an order of magnitude. Serum antiphospholipid and antinuclear antibodies results showed that in the model group antiphospholipid and antinuclear antibodies increased significantly. In the treatment group, antiphospholipid and antinuclear antibodies decreased significantly. This result indicates that UC-MSC therapy is effective in the SLE model. Urine protein quantitation results also showed that UC-MSC therapy can effectively decrease urine protein concentrations in the SLE model.

In summary, we reviewed a large body of domestic and foreign literature and established a tree shrew SLE model. The pathological and serological results and genomics showed that the SLE tree shrew model was established successfully. After initial UC-MSC therapy, we found labeled cells in the heart, liver, spleen, lung, and kidney of tree shrews in the treatment group. The region of interest (ROI) fluorescence values were higher than in the control tree shrews, and most of these ROI values were larger by one order of magnitude. These findings provide a theoretical and experimental basis for using UC-MSCs to treat SLE.

## Conclusions

Ten modeled tree shrews from the pristine and LPS injection groups were grouped into the treatment group and the model control group, with five animals per group. Five normal tree shrews were selected as the normal control group. DiR-labeled UC-MSCs (1 × 10^6^ cells) were transplanted into animals of the treatment and normal control groups. Two weeks after transplantation, the heart, liver, spleen, lung, and kidney of the three groups were stained with HE and kidney Masson and PAS stain, and were imaged. The results revealed obvious pathological changes in the model group, while the treatment group returned to normal levels. The imaging results showed that labeled cells were mainly localized in the lungs, liver, and spleen in the treatment group, and the ROI value of organs of the treatment group was significantly higher than in the normal control group and in the model control group. This difference was statistically significant (*P* < 0.05). This finding proved that UC-MSCs have a therapeutic effect in the tree shrew SLE model. In the future, we can use UC-MSCs to treat human SLE in clinical trials.

## Additional file

Additional file 1: Presents the methods for quantitative PCR (three steps in total). (DOCX 17 kb)

## Abbreviations

BM-MSC: Bone marrow mesenchymal stem cell
EAE: Autoimmune encephalomyelitis
IL: Interleukin
LPS: Lipopolysaccharide
MSC: Mesenchymal stem cell
OD: Optical density
RA: Rheumatoid arthritis
SLE: Systemic lupus erythematosus
Th17: T-helper 17
TNFα: tumor necrosis factor alpha
UC-MSC: Umbilical cord mesenchymal stem cell
