# Supplementary material for: Establishing a tree shrew model of systemic lupus erythematosus and cell transplantation treatment
Source: Stem Cell Res Ther. 2016 Aug 24;7(1):121. doi: 10.1186/s13287-016-0385-1 (PMC4995612; doi:10.1186/s13287-016-0385-1)
Supplement: Additional file 1: — Presents the methods for quantitative PCR (three steps in total). (DOCX 17 kb) [file 13287_2016_385_MOESM1_ESM.docx]

**The supplementary information**

**Quantitative PCR：Three steps in total**

**Blood RNA extract steps：**

Procedure：

Note: Add the proportion volume of 100% ethanol to the bottles labeled Wash Buffer WB and 70% ethanol based on the instruction in the bottles before starting.

1. Pipet 750μL Lysis Buffer RLS to a 1.5mL microcentrifuge tube. Add 250μL whole blood, biological fluids, or other liquid samples to the Lysis Buffer RLS in the microcentrifuge tube. Vortex for 2min to mix thoroughly.

If the sample volume is larger than 250μL, increase the amount of Lysis Buffer RLS proportionally (e.g., a 500μL sample will require 1500μL Lysis Buffer RLS).

Generally, plasma, serum, and other body fluids often have low RNA. Hence, to concentrate these samples is recommended. Please follow the manufacturer’s instructions to concentrate samples to the final volume 250μL.

1. Incubate for 10min at RT.
2. Add 150μL chloroform and shake tubes vigorously for 15sec, and incubate for 3min at RT.
3. Centrifuge the samples at 12,000 rpm for 10min at 4℃.The mixture separates into 3 phases: an upper aqueous phase, interphase and a lower phenol-chloroform phase. RNA remains in the upper aqueous phase. The volume of aqueous phase is around 60% of Lysis Buffer RLS for homogenization.
4. Transfer the aqueous phase to a fresh tube, add 500μL 70% ethanol.
5. Place the Spin-column AC to the Collection Tube, transfer the alcohol-aqueous mixture to the Spin-column AC, centrifuge at 10,000 rpm for 1min, and discard the filtrate.
6. Place the Spin-column AC back to Collection Tube; add 500μl Protein Precipitation Buffer RE to Spin-column AC, centrifuge at 10,000 rpm for 1min, and discard the filtrate.
7. Place the Spin-column AC back on the Collection Tube, add 700μl Washing Buffer RW, centrifuge at 12,000 rpm for 1min, and discard the filtrate.
8. Add 500μl Washing Buffer RW, centrifuge at 12,000 rpm for 1min, and discard the filtrate.
9. Place the Spin-column AC to the Collection Tube and spin for 2min to remove the residual fluid.
10. Place the Spin-column AC to a 1.5ml RNase-free centrifuge tube. Add 50-80μl RNase-free water (pre-heated to 65℃-75℃ is better) to the center of the Spin-column AC. Leave it at room temperature for 2min. Centrifuge at 12,000rpm for 1min. If desired, wash the Spin-column AC with 30μl RNase-free water, combining the second eluate with the first in the same tube; approximately 90% of the RNA is recovered during the first elution step.

**Reverse transcription steps:**

| ddH_2_O | 8ul |
| --- | --- |
| 5×buffer | 4 ul |
| Olig dT | 1 ul |
| inhibitor | 1 ul |
| MLV | 1 ul |
| 10mMdNTP | 2 ul |
| RNA | 3 ul |

42℃60min，70℃10min。

**Quantitative PCR steps:**

| mix | 10 ul | 100 |
| --- | --- | --- |
| Upstream primer | 0.4 ul | 4 |
| Downstream primer | 0.4 ul | 4 |
| ddH_2_O | 6.2 ul | 62 |
| cDNA | 3 ul（20 ul cDNA +80 ul ddH_2_O） | 17ul mixture per tube，add 3ulcDNA |

Condition:

50 degrees 2 minutes

95 degrees 10 minutes

95 degrees 15 seconds

60 degrees 30 seconds

72 degrees 30 seconds, 40 cycles

65 degrees, up 0.5 degrees every 5 seconds, until the rise to 95 degrees (Dissociation Stage)

The relative expression of IL-17 and Foxp3 was normalized by comparison to GAPDH.
